# PYM: a new, affordable, image-based method using a Raspberry Pi to phenotype plant leaf area in a wide diversity of environments

**DOI:** 10.1186/s13007-017-0248-5

**Published:** 2017-11-08

**Authors:** Benoît Valle, Thierry Simonneau, Romain Boulord, Francis Sourd, Thibault Frisson, Maxime Ryckewaert, Philippe Hamard, Nicolas Brichet, Myriam Dauzat, Angélique Christophe

**Affiliations:** 1 UMR759 Laboratoire d’Ecophysiologie des Plantes sous Stress Environnementaux (LEPSE), INRA, Montpellier SupAgro, 2 Place Pierre Viala, 34060 Montpellier Cedex 2, France; 2 Sun’R SAS, 7 rue de Clichy, 75009 Paris, France; 3 Sun’R SmE, 7 rue de Clichy, 75009 Paris, France

**Keywords:** Image analysis, Leaf area measurement, Low cost phenotyping, PYM (raspberry Pi pYthon iMaging), Field phenotyping, Raspberry Pi, Infra-red camera

## Abstract

**Background:**

Plant science uses increasing amounts of phenotypic data to unravel the complex interactions between biological systems and their variable environments. Originally, phenotyping approaches were limited by manual, often destructive operations, causing large errors. Plant imaging emerged as a viable alternative allowing non-invasive and automated data acquisition. Several procedures based on image analysis were developed to monitor leaf growth as a major phenotyping target. However, in most proposals, a time-consuming parameterization of the analysis pipeline is required to handle variable conditions between images, particularly in the field due to unstable light and interferences with soil surface or weeds. To cope with these difficulties, we developed a low-cost, 2D imaging method, hereafter called PYM. The method is based on plant leaf ability to absorb blue light while reflecting infrared wavelengths. PYM consists of a Raspberry Pi computer equipped with an infrared camera and a blue filter and is associated with scripts that compute projected leaf area. This new method was tested on diverse species placed in contrasting conditions. Application to field conditions was evaluated on lettuces grown under photovoltaic panels. The objective was to look for possible acclimation of leaf expansion under photovoltaic panels to optimise the use of solar radiation per unit soil area.

**Results:**

The new PYM device proved to be efficient and accurate for screening leaf area of various species in wide ranges of environments. In the most challenging conditions that we tested, error on plant leaf area was reduced to 5% using PYM compared to 100% when using a recently published method. A high-throughput phenotyping cart, holding 6 chained PYM devices, was designed to capture up to 2000 pictures of field-grown lettuce plants in less than 2 h. Automated analysis of image stacks of individual plants over their growth cycles revealed unexpected differences in leaf expansion rate between lettuces rows depending on their position below or between the photovoltaic panels.

**Conclusions:**

The imaging device described here has several benefits, such as affordability, low cost, reliability and flexibility for online analysis and storage. It should be easily appropriated and customized to meet the needs of various users.

**Electronic supplementary material:**

The online version of this article (10.1186/s13007-017-0248-5) contains supplementary material, which is available to authorized users.

## Background

Crop breeding is considered as a major workaround to feed the growing world population, with a 9–10 billion people forecast by 2050 [1]. Researchers and breeders therefore looked after relevant plant traits to improve crop yield [2]. Plant biomass logically predominated as a target trait directly related to net primary production. Several techniques have been developed to phenotype plant biomass with variable accuracy, easiness of use and cost [3, 4]. The most straightforward method remains plant harvesting and weighing. However, besides its time and labour cost, this procedure is destructive and not compatible with analysis of growth dynamics on individual plants. Yet, sequential monitoring of individual plants proved to be efficient to unravel complex interactions between genotype and environment and to decipher genetic determinism of plant growth submitted to environmental constraints [5, 6]. Non-destructive methods for automated plant phenotyping (http://www.plant-image-analysis.org) therefore received growing interest [7–17].

Plant imaging allows fast, non-invasive phenotyping to dynamically infer plant growth at high throughput [18]. It has benefited from recent advances in navigation, industrial automation and medical diagnostic techniques [4]. Several methods combining image capture and analysis have been proposed with successful applications [19–28] but most were developed for specific environments. Since none of them elicited unanimity, their use in broad ranges of environmental conditions can be questioned. Most often manual and time-consuming parameterization of the image analysis process is required to correctly discriminate the plant from its background when leaf colour, light environment and background conditions are not stable [12]. In some cases, re-parameterization is even not affordable since analysis has been implemented in non-publicly available software [6, 8, 27].

Rather than looking for sophisticated analysis of standard images, stepping back and adapting the hardware to capture more suitable images can simplify the analysis and extend the domain of application. Usual detection of plant leaves on standard images relies on the ability of photosynthetic tissues to reemit visible light (VIS; 400–700 nm wavelengths) in specific, mostly green wavelengths which are not absorbed by leaf pigments [29]. Imaging plants in an extended spectrum, including wavelengths where leaves exhibit specific spectral properties, could open new perspectives. In particular, leaf tissues re-emit solar radiation in the near infrared wavelengths (NIR; 700–1100 nm) [29]. These properties gave rise to the development of the Normalized Difference Vegetation Index (NDVI, [30]), initially based on images collected by the satellite Landsat 1 equipped with a multispectral scanner. NDVI compares red and NIR reflectances and ranges from negative values corresponding to non-vegetative soils to positive values, comprised between 0.1 and 0.7 and typical of plant covers [31]. It has been correlated with several traits such as vegetation coverage [32], green biomass [33], nitrogen content [34, 35] and grain yield [36]. NDVI has been implemented in portable commercial solutions for field phenotyping (for example Greenseeker™, [37]) but can also be derived from any camera able to detect signals in infrared (IR) or NIR wavelengths. High resolution, hyperspectral cameras are the most flexible ones as regards separation of specific wavelengths enabling to detect plant stress [11, 13], but they remain quite expensive. By contrast, standard cameras are now available at very low cost but are equipped with infrared blocking filters to limit image capture in the VIS range.

Here we examined how the spectral characteristics of plant leaves could be included in a low cost, portable and automated imaging system to determine isolated plant leaf area in a wide range of conditions. We describe such an efficient solution using the widely spread Raspberry Pi [38] computer with a modified version of a standard camera module (Pi NoIR) where the IR filter was removed to extend light capture beyond the VIS range. A blue filter (provided by the manufacturer) was also added. Scripts were developed for the resulting images to determine the projected leaf area of plants. We demonstrate that plant segmentation with this new device is efficient for various species and background environments, while standard methods often fail to correctly estimate plant leaf area. Reliability in field conditions is illustrated by data obtained on lettuce plants grown below different configurations of photovoltaic panels (PVPs). The concept of growing plants in the partial shade of PVPs emerged in 1982 [39] to cope with the detrimental impacts of climate change on plants and increase global land productivity [40]. At first glance, shading crops with photovoltaic panels is thought to severely hamper plant growth. However, an increase in plant efficiency to intercept radiation (radiation interception efficiency, RIE) has been reported for lettuce grown below PVPs due to the acclimation of leaf expansion to shade and resulting in growth maintenance [41]. To get insights into these acclimation processes, we developed a field-phenotyping cart where several Raspberry Pi devices were chained to monitor leaf area for hundreds of lettuce plants grown below different configurations of PVPs. Compared to full sun conditions, lettuces grown at the vertical of free spaces separating PVPs had enhanced expansion rate of their projected surface and thus increased RIE. However, this acclimation of plants to shading conditions was not sufficient to maintain biomass at harvest. By contrast, plant biomass was closer to that observed in full sun conditions when lettuces were grown at the right vertical below PVPs, where expansion rate of plant surface and thus radiation interception was not significantly altered.

## Methods (can also be placed after Conclusions)

### Image acquisition system

**Fig. 1:**
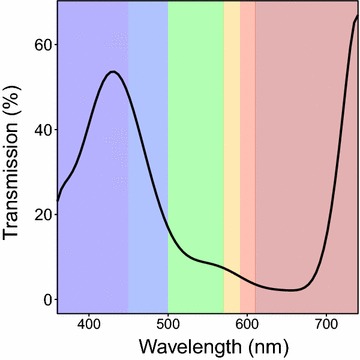
Spectral properties of the blue filter and transmitted wavelengths to the camera sensor. Roscolux #2007 Storaro Blue transmission spectrum. Blue filter stops green and red wavelengths, while blue and infrared are transmitted to the camera sensor

A fully programmable, infrared camera system was built assembling a compact, single-board computer (Raspberry Pi 2 model B) and an infrared camera (Raspberry Pi NoIR V1). The computer was run under Raspbian GNU/Linux operating system and scripts were developed in Python language to facilitate image capture automation and analysis. The camera was a regular module (OmniVision OV5647) where the infrared filter was removed, allowing for the capture of NIR wavelengths in addition to standard VIS light. A blue filter (Roscolux #2007 Storaro Blue) was also set in front of the camera lens to exclude green and red wavelengths and to transmit blue and NIR wavelengths higher than 700 nm (Fig. 1). Overall, the light incoming to the camera lens was mainly composed of VIS light filtered for blue and NIR wavelengths which were recorded in the BLUE and RED channels, respectively.

A standard camera module (Raspberry Pi) equipped with its original infrared filter was also used to compare with other methods in controlled conditions. Source images were designed as VIS images when captured with this standard camera or (VIS + NIR)_BF_ images when captured with the modified one as described above.

### Image capture and analysis

#### Overall description

Software for the automation of image capture, segmentation and analysis was developed in Python 2.7 (http://www.python.org).

A first python script was developed and uploaded in each Raspberry Pi to control image capture and storage in USB flash disks. Image analysis was performed with another, specifically developed python script based on Numpy (http://www.numpy.org) and OpenCV2 (http://opencv.org) libraries. This step could be performed in Windows operating systems via an executable programme using py2exe (http://www.py2exe.org). The script can batch process thousands of pictures in a few minutes on a standard personal computer, including storage of final images for rapid control of the procedure and saving the end results (leaf area) directly into a spreadsheet-ready CSV file.

#### Segmentation method

The first step of image analysis was the transformation of the original picture into a new one using selected wavelengths specific to leaves. Leaf emission spectrum is largely determined by the photosynthetic pigments, mainly chlorophylls and carotenoids. As a consequence, most species exhibit green leaves, due to pigments absorbing blue and red regions in the VIS [42]. However, these properties do not discriminate efficiently against many backgrounds. By contrast, the internal cellular structure of leaf cells is more specifically responsible for a high reflectivity of near-infrared light [43]. Using the plant ability to absorb blue light and reflect near-infrared light, we developed a method able to extract leaf surface from its background. The source image needs to be taken with the camera system described above, associating the infrared-transformed camera and a blue filter. Colour image recording is usually split into BLUE, GREEN and RED channels corresponding to the output format of the camera (raw RGB). RED (mainly encompassing NIR wavelengths) and BLUE channels were sufficient to segment the plant from its background in our procedure. High intensity in the RED channel and low intensity in the BLUE one coincided with the presence of vegetation reflecting near-infrared wavelengths whereas near-infrared reflection was negligible for most other material around plants. By subtracting pixel values of BLUE channel to that of the RED one, non-vegetative pixel values were further attenuated, increasing the contrast between plant and background. A new image was thus created by subtracting the BLUE to RED channel intensity at each pixel according to Eq. (1).

1 $${\text{NEW}}\;{\text{CHANNEL}} = {\text{RED}} - k \times {\text{BULE}}\; \left({{\text{with}}\;{\text{k}} = 0.5} \right)$$

k is a fixed parameter whose value was optimized empirically. Higher values (closer to 1) resulted in low intensity images with substantial segmentation errors while lower values (closer to 0) hindered the discrimination when background areas were detected in the RED channel. Pixel intensities in the different channels were coded as unsigned 8-bit integers, assigning to zero any negative value of NEW CHANNEL which corresponded to non-vegetative pixels with high intensity of BLUE. Intensity of pixels was rescaled after NEW CHANNEL computation.

**Fig. 2:**
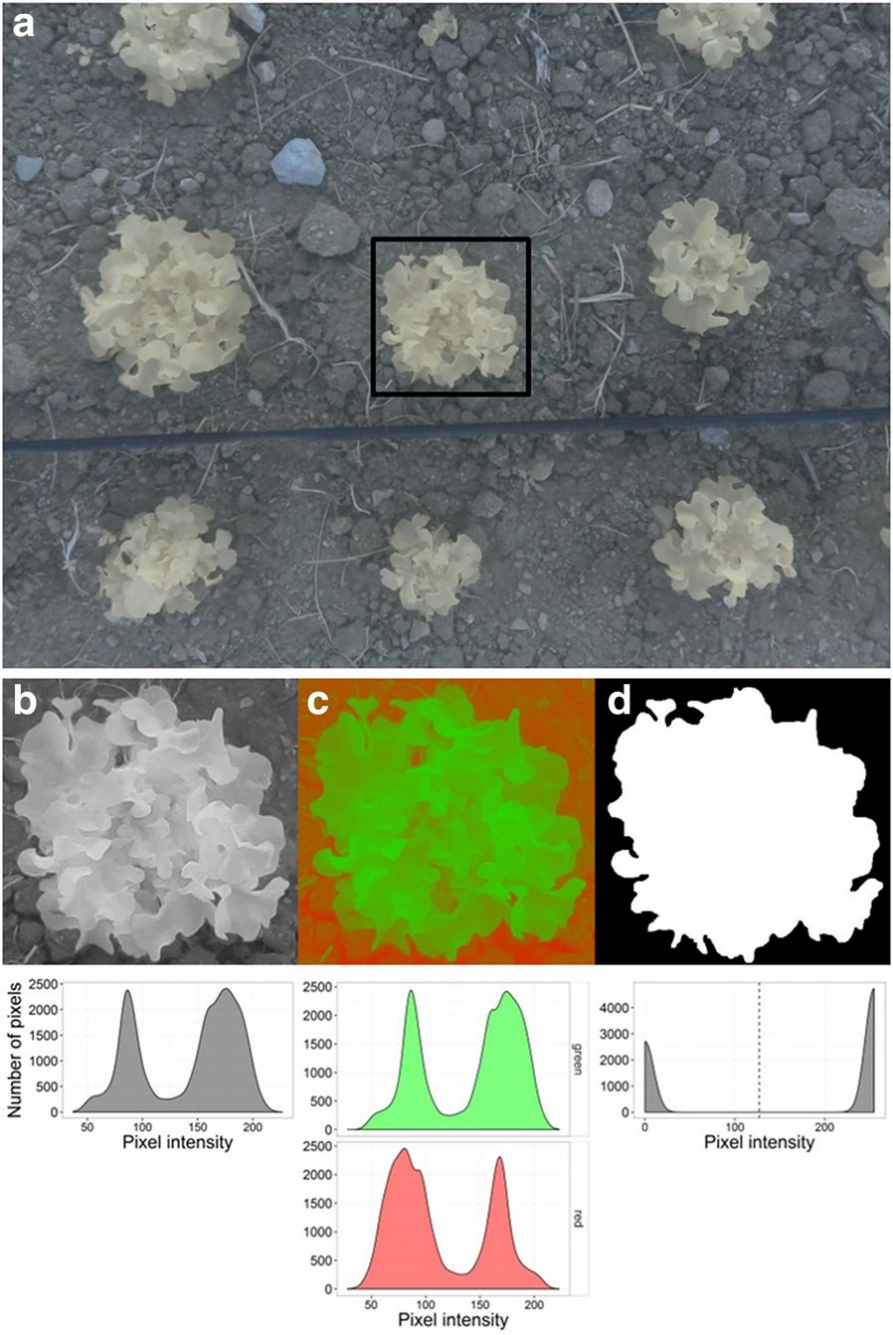
Details of image analysis process used in the PYM procedure, from source to segmented image (**a**–**d**). **a** Source image taken with the infrared camera and the blue filter. **b** Grey image based on NEW CHANNEL values (after a crop of a image). **c** False 2 colour image for visual assessment of segmentation between plant and background. **d** Final image after segmentation, erosion, opening and closing procedures. White pixels are counted and summed to estimate projected leaf area

Application of Eq. (1) resulted in a narrow range of variations in pixel intensity for this NEW CHANNEL (Fig. 2b). The distribution of pixel intensity in this new channel is bimodal with each mode associated with either the plant or the background prefiguring the segmentation.

A false 2 colour image based on this NEW CHANNEL image can be created at this stage (included in our script but may be skipped) to better visualize the segmentation between plant and background (Fig. 2c). An adaptive thresholding based on Otsu’s binarization [44] was then processed on the NEW CHANNEL image (Fig. 2b).

Standard procedures for erosion, opening and closing were then applied in that order to remove noise and closing small holes inside objects using OpenCV library. Contour of each object in the scene was then drawn and individual areas calculated. When plants were isolated, only one object was detected. In some cases, small additional objects like weeds were counted and were automatically disregarded by assigning the highest area in the picture to the plant surface (Fig. 2d). In the case of measures in controlled conditions and for plants with no contiguous contour, plant leaf area was computed as the sum of all white pixels. The result was stored as pixel number and converted into cm^2^, using a conversion ratio measured with a calibration standard placed at the soil level in the field of view using PYM cameras.

### Segmentation performance assessment

We compared our proposed PYM (raspberry Pi pYthon iMaging) procedure with Rosette Tracker, a state-of-the-art, recently published method. Rosette Tracker emerged as the only published method able to estimate plant leaf area, freely available as an ImageJ plugin with minimal parameterization of the analysis software, thereby sharing similar objective as PYM, although working on VIS images [12]. We therefore retained two different versions of the same camera, either standard for imaging in VIS wavelengths or transformed for (VIS + NIR)_BF_ imaging as above described. Paired pictures were obtained with both cameras from various scenes combining leaves or whole plants of various species over different backgrounds. Automated segmentation of VIS and (VIS + NIR)_BF_ images were performed with their respective method (Rosette Tracker or PYM).

As a reference, manual segmentation of both original images (VIS and (VIS + NIR)_BF_) was operated with ImageJ, drawing precise contours of each leaf or plant to determine their areas with maximal accuracy using a high resolution tablet (2560 × 1440 pixels, Wacom, Germany). First, each object was drawn and filled using the *Brush* tool in ImageJ. The VIS and (VIS + NIR)_BF_ images were then transformed to 8 bit (greyscale) and then thresholded to produce binary images.

For the 4 types of segmented images (on (VIS + NIR)_BF_ images using PYM or manual segmentations and on VIS images using Rosette Tracker or manual segmentation), the *Measure* tool in ImageJ provided the object area as a number of pixels. For each scene, the reference leaf or plant area was computed as the mean of the 2 areas determined by manual segmentation of VIS and (VIS + NIR)_BF_ images. The performance of the two segmentation methods (Rosette tracker and PYM) was evaluated by comparing leaf area generated by each automated method to this reference area.

To compare methods in standard conditions for Rosette Tracker, 149 pre-bolting *Arabidopsis thaliana* plants (several genotypes) were photographed in the PHENOPSIS high-throughput phenotyping platform [45]. Two successive sequences of photographs were shot with the two types of camera VIS and (VIS + NIR)_BF_.

**Fig. 3:**
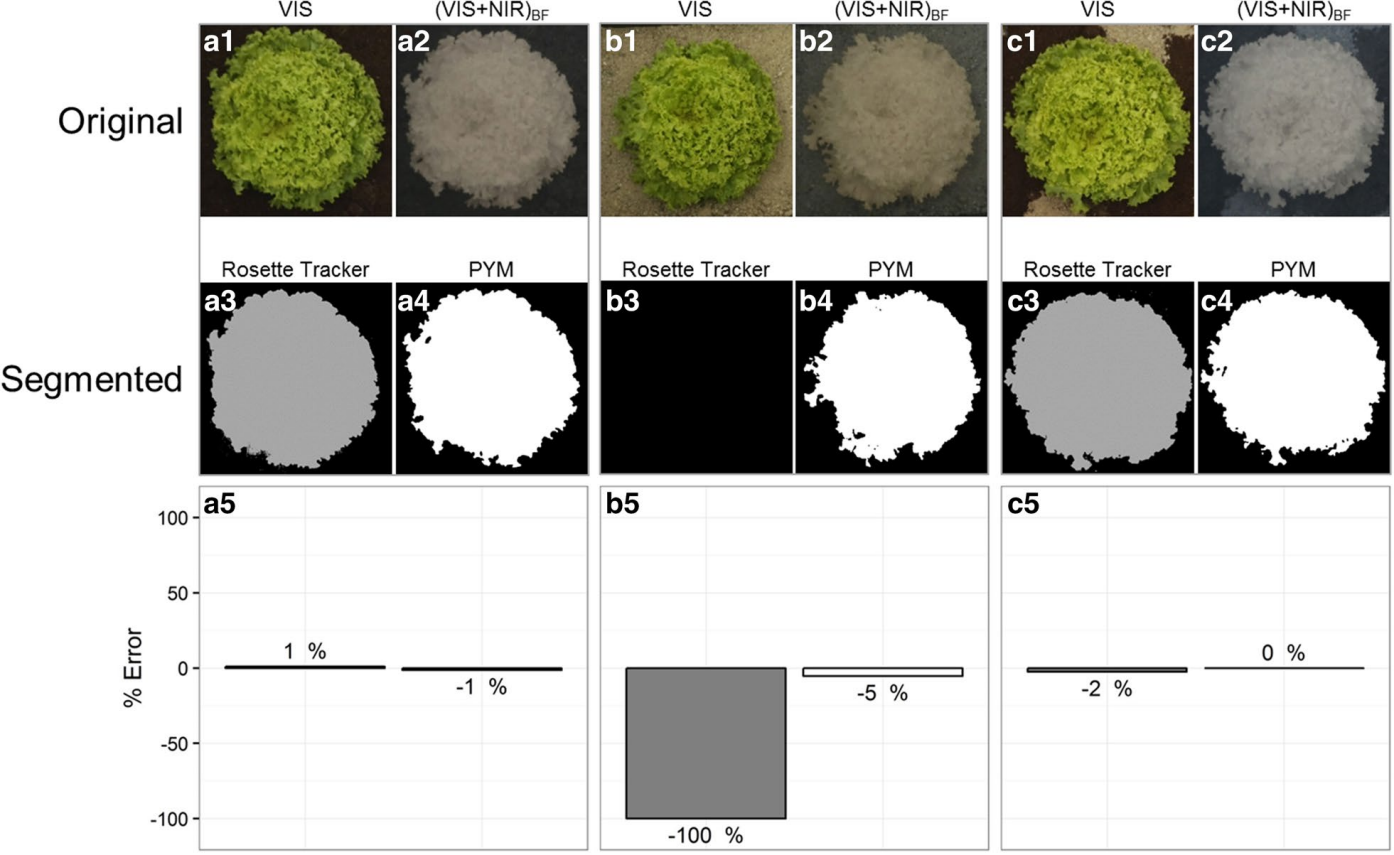
Performance of the PYM segmentation method with contrasted background conditions. The same lettuce was photographed with three different soil backgrounds. The PYM method was compared to Rosette Tracker developed on VIS images [12]. **a1**–**c1** VIS image. **a2**–**c2**: (VIS + NIR)_BF_ image. **a3**–**c3** Segmented image after Rosette Tracker procedure. **a4**–**c4** Segmented image after PYM procedure. **a5**–**c5** Error (%) made on plant leaf area using automated procedures relative to the reference area determined on manually contoured plant on original images

**Fig. 4:**
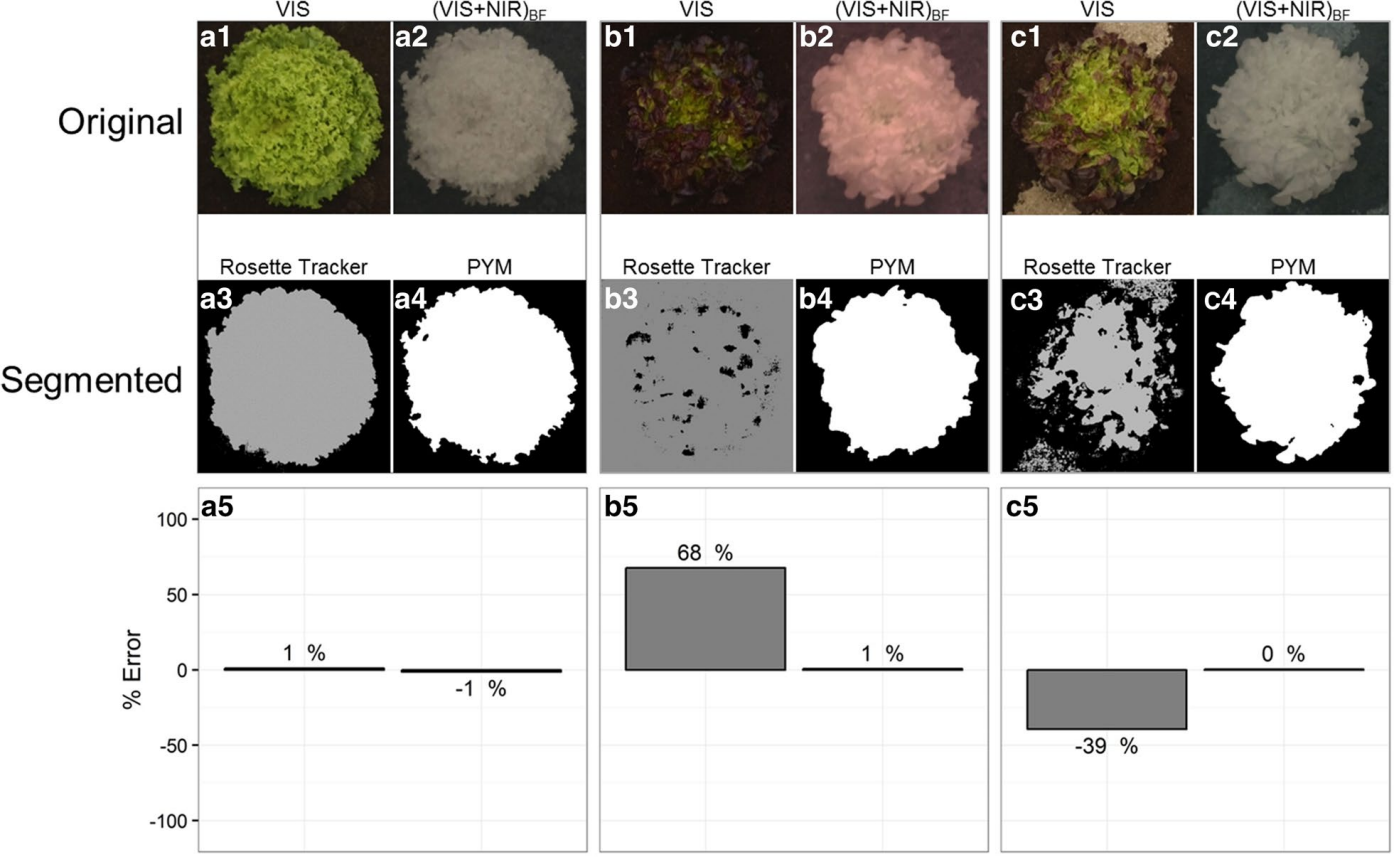
Performance of the PYM segmentation method with contrasted varieties of lettuce (light green in A or dark red in B and C). Comparison with Rosette Tracker [12]. **a1**–**c1** VIS image. **a2**–**c2** (VIS + NIR)_BF_ image. **a3**–**c3** Segmented image after Rosette Tracker procedure. **a4**–**c4** Segmented image after PYM procedure. **a5**–**c5** Error (%) made on plant leaf area using automated procedures relative to the reference area determined on manually contoured plant on original images. Panel **a** is a duplicate of Fig. 4 for comparison purposes

To compare methods in various, challenging situations, dark soil, clear sand and a combination of both substrates were associated with two lettuce varieties: a green one (frilly lettuce) and a red one (red oak leaf lettuce), bought in retail and placed over different backgrounds under artificial light (Figs. 3, 4).

### Application to field experiment

In summer 2015, an experiment was carried out with lettuces (Kiribati variety, Oakleaf, *Lactuca sativa acephala sp.*) grown in a field at Montpellier, France (43°6N, 3°8E). Lettuces were planted in boards of 6 rows, with 30 cm distance between rows and between plants within a row. Irrigation was provided by drip lines so as to ensure absence of water stress. Macronutrient fertilization was applied the day before planting and nitrogen status was then weekly controlled with a chlorophyllmeter (SPAD-502, Konica Minolta Inc., Japan) to verify that nitrogen was not limiting.

Growth of individual plants was followed in different light conditions imposed by photovoltaic panels (PVPs) installed over the crop in addition to full sun conditions as a control. A similar experiment was previously carried out with fixed PVPs [41]. In the present study, we analysed a new system equipped with solar trackers which could move around North–South horizontal axes to track the daily course of the sun with technical limits of − 50 and + 50 degree angles with horizontal. They were programmed to adjust their position every time a 1 degree offset was detected between sun azimuth and the direction normal to panels so as to maximize interception of solar radiation. Photovoltaic panels were joined into 2 m wide and 19 m long, North–South strips and placed 5 meters above ground. PVP density was adapted to crop needs by leaving 4.4 m wide [40], free spaces separating each PVPs strips (with horizontal panel orientation). The whole system generated strips of shade and sun at soil level moving from West to East throughout the day.

Lettuces were planted on 21th of July 2015. Two plots were cropped under photovoltaic panels. The first one was located at the vertical below the PVPs (Below_PVPs treatment) and the second one below the free space leaved between two strips of PVPs (Between_PVPs treatment). As a consequence, plants of the two treatments were shaded at different periods of the day although they received quite similar levels of radiation over the whole day (see “Results”). The control (full sun) plot, where plants received much higher radiation, was positioned at the South of the two PVP treatments to prevent undesired shading by the panels.

In order to characterise time changes of projected leaf area of plants at high-throughput, a set of 6 modified PYM devices was mounted onto a phenotyping cart (see Additional file 1). To fit the plantation design, the cart consisted of a light, metallic structure equipped with wheels so that it could be translated spanning over the 6 rows of the plantation boards. A camera was associated with each row, resulting in 6 cameras spaced by 30 cm as were the rows of lettuces. Cameras were fixed to a horizontal rod stepping at 1 m above the crop. Raspberry Pi computers (one per camera) were connected through GPIO pins to a single contactor triggering the 6 cameras simultaneously (see Additional file 2). Pictures were directly stored into an USB flash disk. Portable power banks supplied power to the computers. Vertical alignment of the cameras over each line of 6 lettuces normal to planting rows was ensured by positioning the wheels of the cart on the same line as the imaged plants. On sunny days, a tissue shelter was installed on the cart over the cameras to project a uniform shade on the whole field of view of each camera. Throughout the growing period, 30–50 plants per treatment were photographed twice a week resulting in 9 pictures per plant recorded from 6 to 37 days after plantation. All pictures (78 per plot) were taken within 20 min starting at 10:00 a.m. For late developmental stages, overlapping between plants was occasionally detected on some images which required manual contour of individual plant leaf area including estimation of covered leaf surfaces when appropriate.

Plants were harvested 37 days after plantation, at the same date for all treatments, corresponding to the commercial maturity stage for the full sun conditions (i.e. about 400 g fresh weight per plant). For each treatment, 18 plants were collected and rapidly washed to remove soil particles, then oven dried for 72 h at 60 °C to determine individual dry weights of whole plants.

### Microclimate in the field experiment

A temperature and humidity probe (HMP45 AC; Campbell Scientific Inc., UK) and a radiation sensor (BF5; Delta-T Devices, UK) connected to a data logger were positioned in the control plot to monitor air temperature and global and diffuse radiations (400–700 nm). Global and diffuse radiations were used to compute radiative balance at the plant level for the different locations under the PVPs by applying a ray tracing algorithm [46] to a three-dimensional numerical representation of the whole photovoltaic system. Instantaneous, global incident radiation transmitted at plant level (Radiation_inc_) was thus computed every 3 min taking into account actual changes in sun position and orientation of the photovoltaic trackers. Cumulated, global radiation over the whole growing period was then calculated by integrating these instantaneous values.

### Plant leaf expansion rate and intercepted radiation in the field experiment

The Relative Expansion Rate (RER, [27, 47, 48]) was determined for the projected leaf area of each plant (Plant Leaf Area) at each time interval between two consecutive dates of image capture. Thermal time (TT) was preferred to legal time to remove the effects of temperature variations between days and treatments. TT was calculated as the daily cumulated difference between mean air temperature and a minimal of 3.5 °C required for lettuce growth [49, 50]. RER was thus determined as follows (Eq. 2):

2 $${\text{RER}}_{\text{i}} = \left[{\frac{{ln\left[\left({{\text{Plant}}\;{\text{leaf}}\;{\text{area}}} \right)_{{{\text{i}} + 1}} \right]- ln\left[\left({{\text{Plant}}\;{\text{leaf}}\;{\text{area}}} \right)_{\text{i}}\right]}}{{{\text{TT}}_{{{\text{i}} + 1}} - {\text{TT}}_{\text{i}}}}} \right]$$

where i and i + 1 represent two consecutive imaging dates.

Intercepted radiation (Radiation_int_) was estimated for each plant as the product of Plant leaf area with global incident radiation (Radiation_inc_) determined at plant level as above described. Plant leaf area, i.e. the projected leaf area of the plant determined with PYM was considered as a relevant proxy for the surface intercepting solar radiation at the whole day scale due to the hemispherical shape of the lettuces. During most of the growth cycle, plants did not overlap. For late developmental stages, when Plant leaf area exceeded the 30 by 30 cm square dedicated to each lettuce at plantation, a correction was applied to remove overlapping leaf areas between neighbouring plants. The correction consisted in considering the plant surface as a disk of equivalent area to that determined with PYM, from which all surfaces outside a concentric, 30 × 30 cm square were subtracted, which occurred when the radius r of the disk equivalent to the plant exceeded 15 cm. The Plant Leaf Area of such plants was therefore corrected as follows (Eq. 3).

3 $${\text{Plant}}\,{\text{leaf}}\;{\text{area}} = 8 \times \left({\frac{{15 \times {\text{r}} \times { \sin }\left({{\text{arccos}}\left({\frac{15}{\text{r}}} \right)} \right)}}{2}} \right) + 4 \times \left({\frac{\uppi}{4} - {\text{arccos}}\left({\frac{15}{\text{r}}} \right)} \right) \times {\text{r}}^{2}$$

To cumulate the intercepted radiation throughout the growth cycle, the mean Plant Leaf Area for each period between two consecutive imaging dates was multiplied by the incident radiation cumulated during the corresponding period (Eq. 4).

4 $${\text{Radiation}}_{int} = \sum\limits_{i = 1}^{9} {\left[{\frac{{\left({{\text{Plant}}\;{\text{leaf}}\;{\text{area}}\left({i - 1} \right) + {\text{Plant}}\;{\text{leaf}}\;{\text{area(}}i )} \right)}}{2} \times \sum\limits_{t = time(i - 1)}^{time(i)} {{\text{Radiation}}_{inc} (t)\Updelta t}} \right]}.$$

where i represents each imaging date. Plant leaf area at planting (i = 0) was estimated at 10 cm^2^ as an average determined with the PYM method on a subset of plants from all treatments.

Mean efficiency for radiation interception by plants over their whole growth period (RIE) was simply calculated as the ratio of cumulated intercepted radiation to cumulated incident radiation. RIE mainly depended on the dynamics of plant leaf expansion and spatial arrangement of the leaves.

Finally, mean radiation use efficiency by the plant over the whole growth period (RUE) was then deduced as the ratio of accumulated dry mass (determined at harvest date) to cumulated, intercepted radiation (derived from Eq. 4). RUE integrates all the physiological mechanisms involved in the transformation of the radiation intercepted by the plant into harvested biomass.

### Statistical analyses

Light treatment effects on plant traits were analysed through analyses of variance (ANOVAs) and Kruskall-Wallis tests for multiple comparisons. Light treatment effects on RER modelling were assessed using a likelihood ratio test. All statistical tests were performed using R 3.3.1 (R Core Team (2016)).

## Results

### Assessment of PYM segmentation performance

#### Image segmentation with contrasted backgrounds

Efficiency of the PYM method was first assessed by comparison with Rosette Tracker, a recently published method for estimating plant leaf area from VIS images. Since Rosette Tracker operates with a regular version of the camera while PYM operates with a modified one, able to sense (VIS + NIR)_BF_ wavelengths, paired, VIS and (VIS + NIR)_BF_ images were captured for different plants using both configurations of the camera. Methods were tested with images of one same lettuce plant placed over three contrasted backgrounds (Fig. 3) and segmentation was run using scripts developed in Rosette Tracker and PYM respectively. Both segmentation methods correctly estimated the projected plant surface area on a dark background with only a 1% deviation compared to the reference area estimated by manual contour of the plant on original, VIS and (VIS + NIR)_BF_ images (Fig. 3a1–a5). Automated segmentation of the plant (light green) placed over a clear background (Fig. 3b1–b5) generated maximal error when using Rosette Tracker on VIS image, due to a general confusion between the plant and the background. By contrast, our method was able to detect the plant surface area with a deviation limited to 5% when compared to the reference area. When both substrates were mixed (Fig. 3c1–c5), error in estimating leaf area was strongly reduced with Rosette Tracker (2%) but was still higher than with our PYM method (less than 0.5%). Overall, Rosette Tracker performed successful segmentation as long as contrasts between plant and background were present in the VIS image. However, when brightness of the background was close to that of the plant, confusion between the two could be total. Conversely, our PYM method could detect the plant in all, tested conditions with a maximal 5% error on leaf area.

#### Image segmentation with contrasted plant pigmentations

To go further in challenging situations, we picked a dark coloured, red lettuce variety (Fig. 4). As previously outlined with light plant and background, when both soil and plant have a dark colour, methods based on VIS images, largely fail to determine which pixel belongs to whom resulting in errors as large as 68% for plant leaf area (Fig. 4b5). For some VIS images (Fig. 4b1), it can even become tricky to trace the plant contour manually. Intermediary results were obtained on VIS images when the plant (and the background) consisted of a mix of clear and dark areas which generally resulted in underestimation of plant leaf area (by 39% in Fig. 4c5). Our PYM procedure performed much better in all these situations with less than 1% error on plant leaf area.

#### Image segmentation in PHENOPSIS high-throughput phenotyping platform

**Fig. 5:**
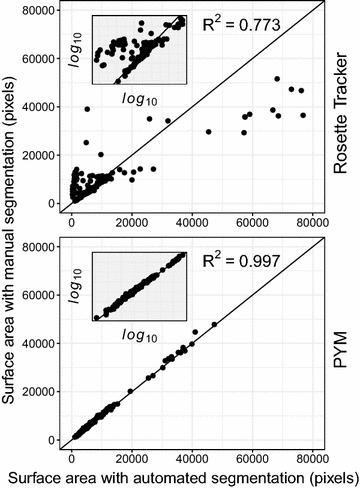
Summary plots of all surface areas determined in parallel with PYM and Rosette Tracker procedures on 149 *Arabidopsis thaliana* plants from different genotypes in the PHENOPSIS platform [45]. Comparison with a reference area determined by manual contour of leaf or plant on original images. Each point derives from one of the 149 photographs

Both segmentation methods were tested in growth chambers conditions in PHENOPSIS, a phenotyping platform for which Rosette Tracker was initially conceived. Different genotypes of *Arabidopsis thaliana* plants were photographed and estimations of plant leaf area with both methods were compared to the manual segmentation of the plant (Fig. 5).

Estimated leaf area tightly correlated with manually determined area when using the PYM method (R^2^ = 0.997). The correlation was much looser (R^2^ = 0.773) with Rosette Tracker working on VIS images with plant leaf area being either over or under estimated. The mean error relative to manually determined leaf areas for all the situations tested was much lower with the PYM method (6.7%) in comparison with the VIS based method Rosette Tracker (34.1%).

#### Extension of the PYM method to various species and conditions

**Fig. 6:**
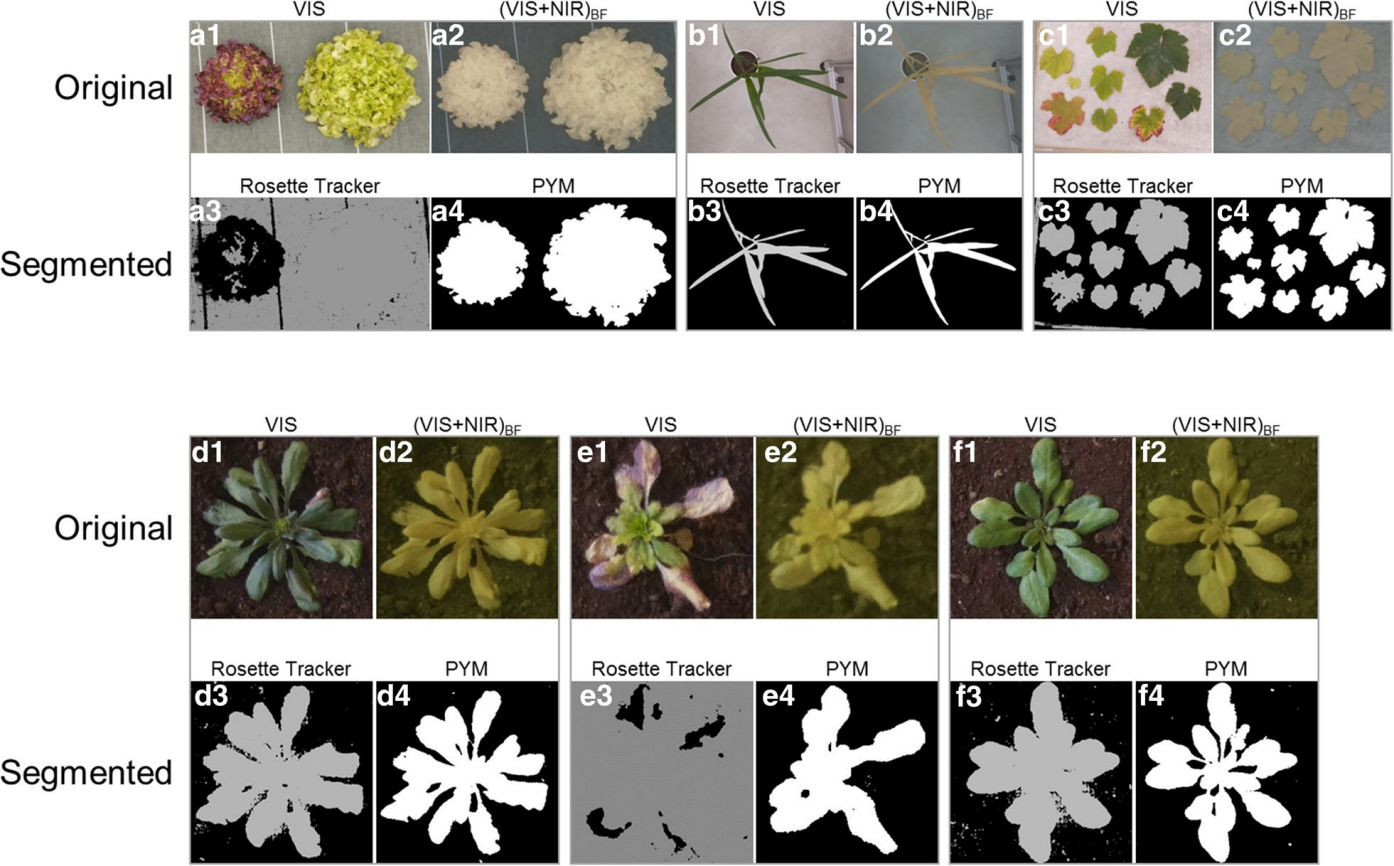
Exploration of the performance of the PYM segmentation procedure with various species in different environments. Comparison with Rosette Tracker [12]. **a** Red and green leaf lettuce placed over plastic cover and analysed together in one same image. **b** Potted maize plant in greenhouse with concrete soil. **c** Several grapevine leaves placed over white table. **d** Arabidopsis thaliana grown in soil with well-watered conditions. **e** Arabidopsis thaliana inoculated with Turnip Mosaic Virus (TuMV). **f** Arabidopsis thaliana grown in soil under water deficit conditions. **a1**–**f1** VIS image. **a2**–**f2** (VIS + NIR)_BF_ image. **a3**–**f3** Segmented image after Rosette Tracker procedure. **a4**–**f4** Segmented image after PYM procedure

Our PYM segmentation method was also tested on different species placed in different growth conditions (Fig. 6). Either Rosette Tracker or PYM well performed when the plant coloration was uniform (Fig. 6b and, to a lesser extent, 6d and f). However, when several leaves of one same plant or several plants in the same image exhibited contrasted colours (Fig. 6a, c, e), only the PYM method was able to retrieve the entirety of the leaf area. Interestingly, all images in Fig. 6 were captured and processed with PYM using a unique device and a stationary script. This opens interesting applications for detecting leaf area of plants with optical variations such as chlorotic or necrotic surfaces (Fig. 6e).

### Application of PYM method to a field experiment

#### Dynamics of leaf area expansion in a field experiment with lettuce plants

**Fig. 7:**
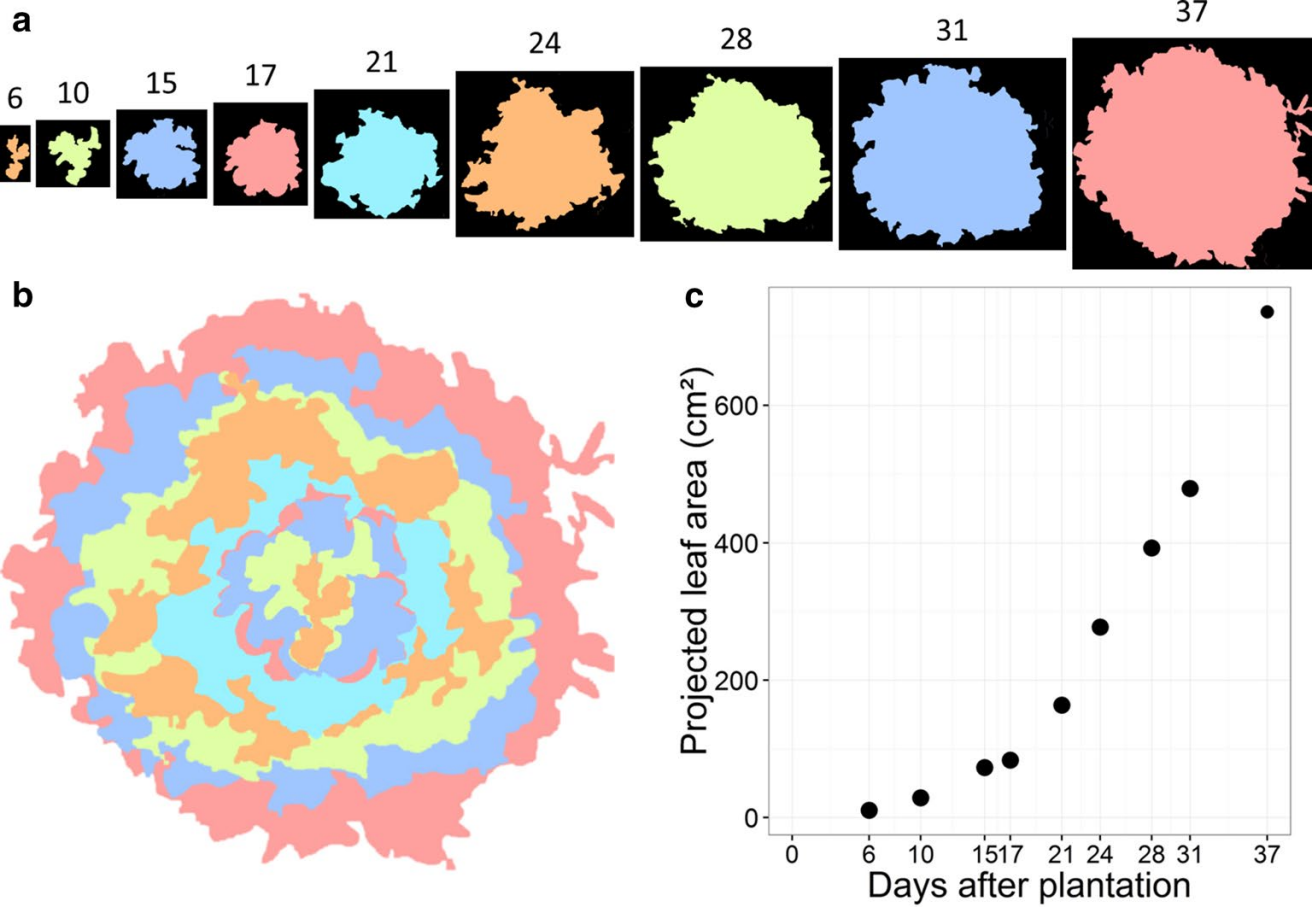
Growth of lettuces cultivated in field conditions. **a** An example of image stack of projected leaf area for a lettuce plant photographed with a phenotyping cart at 9 dates from plantation to harvest in a field experiment performed in summer 2015. Numbers above pictures are Days After Plantation (DAP). **b** Superposition of the processed images of the projected leaf area for one plant (same as in A) with the youngest stage forefront. From the forefront centre to background: orange = 6 DAP, green = 10 DAP, blue = 15 DAP, red = 17 DAP, cyan = 21 DAP, orange = 24 DAP, green = 28 DAP, blue = 31 DAP, red = 37 DAP. The proportions of image sizes are preserved. **c** Evolution of the projected leaf area of the selected plant from plantation to harvest

Three plots of lettuces receiving standard irrigation and fertilization but different light treatments were compared in field conditions. A first plot was submitted to full sun conditions and was considered as the control. A second plot was aligned at the right vertical below a strip of joining, photovoltaic panels (Below_PVPs treatment) and a third plot was placed between two strips of PVPs (Between_PVPs treatment). Dynamics of projected leaf area was determined for 30–51 plants per treatment with a phenotyping cart equipped with 6 PYM devices. Imaging was repeated on the same plants at 9 dates from plantation to harvest. The automated PYM procedure provided enough resolution to monitor small increments in projected leaf area between two consecutive images captures regardless of changes in soil surface and light conditions (Fig. 7).

**Fig. 8:**
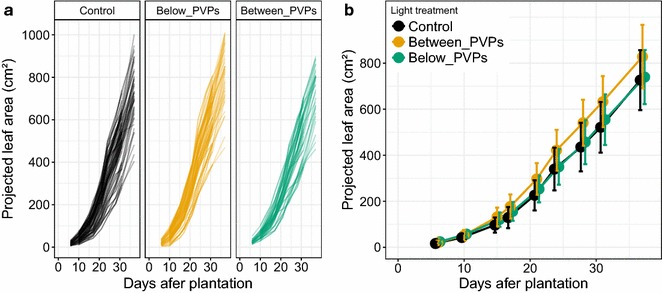
Projected leaf area dynamics for 124 lettuce plants submitted to three different light treatments due to shading by photovoltaic panels (PVPs) placed at 5 m above plants. Control corresponds to full sun condition. In other treatments, plants were grown at the right vertical below a strip of joining photovoltaic panels (Below_PVPs treatment) or between two strips of PVPs (Between_PVPs treatment). **a** Each curve represents the evolution of projected leaf area for one same plant measured at 9 dates from 6 to 37 days after plantation. **b** Same as A except that projected leaf area was averaged for all plants of each treatment on each date. Error bars indicate standard deviation for a minimum of 30 plants

Overall, individual plants had very scattered evolutions of their projected leaf area, including within each light treatment (Fig. 8a). However, by repeating leaf area determination with the phenotyping cart on large sets of plants, a significant difference (*p* value < 10^−3^) in leaf expansion was evidenced across light treatments (Fig. 8b). Projected leaf area was significantly higher for plants grown between PVPs than for the plants placed below PVPs or in full sun conditions. Yet, Between_PVPs and Below_PVPs treatments exposed the plants to quite similar levels of radiation at the whole day scale, although with a different timing for shade and sunlit periods throughout the day. Mean daily radiation at plant level over the whole growth period amounted to 29 and 31 mol m^−2^ day^−1^ for these treatments (Below_PVPs and Between_PVPs respectively) compared to 44 mol m^−2^ day^−1^ for full sun conditions.

#### Relative Expansion Rate in the field experiment

**Fig. 9:**
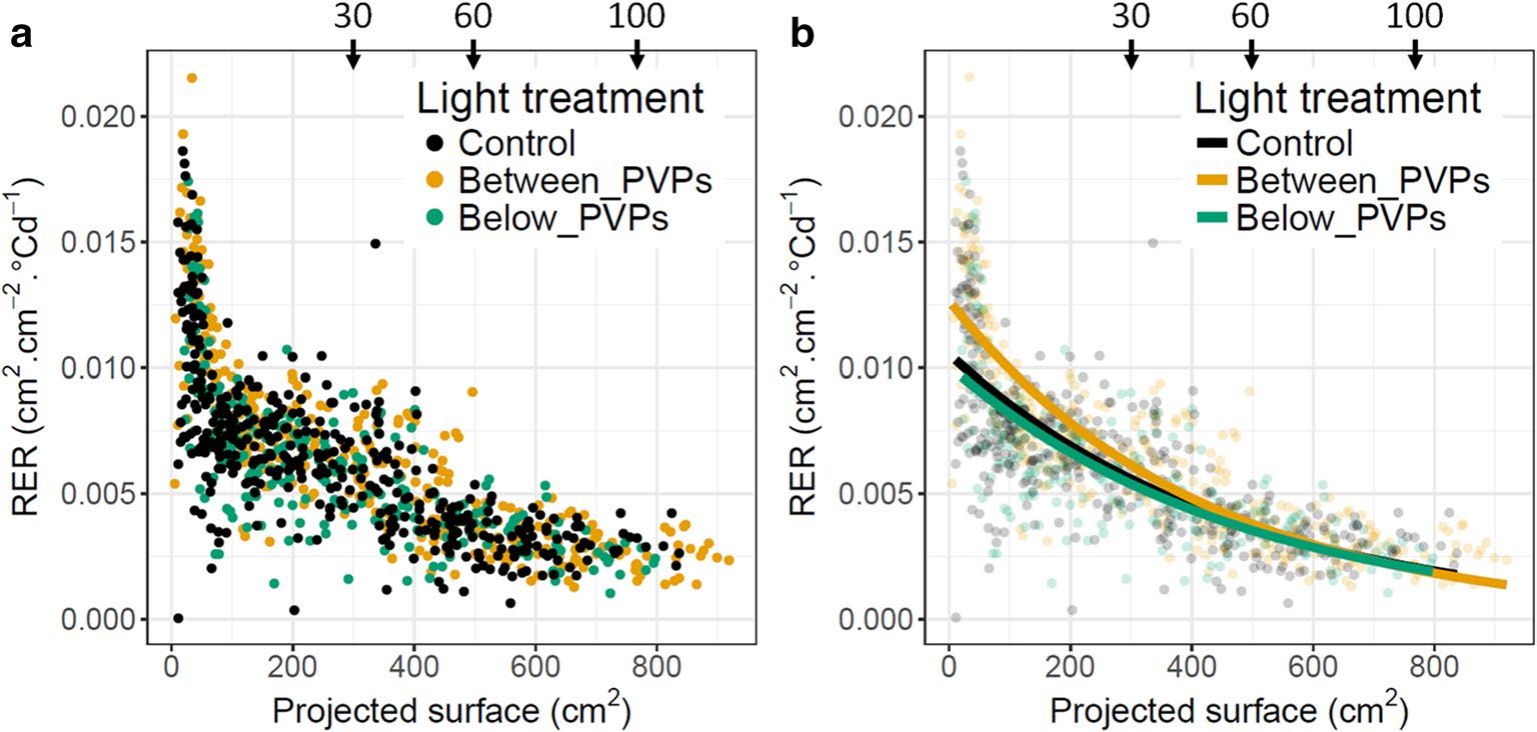
Evolution of Relative Expansion Rate (RER) of lettuce plants grown in field conditions with different light treatments as a function of projected leaf area. RER was calculated for individual plants and each time interval between two consecutive dates of image capture. Same notation as in Fig. 10. Control corresponds to full sun condition. In other treatments, plants were grown at the right vertical below a strip of joining photovoltaic panels (Below_PVPs) or between two strips of PVPs (Between_PVPs). Black arrows correspond to leaves number (approx.). **a** RER was computed as the local slope of the relationship between the natural logarithm of projected leaf area and thermal time. X axis represents the mean projected leaf area between 2 image captures. **b** Same as A with lines corresponding to the following equation: y = exp(α + β * x)

For each plant, relative expansion rate (RER) of individual plants was computed for the 8 periods separating 2 consecutive image captures. A general decline was evidenced for RER when related to the projected leaf area considered as the mean of initial and final values for each time interval (Fig. 9a). This indicated that RER was largely determined by the plant surface able to intercept light at each time interval, although with a tendency to decline with plant development for all three treatments. This corresponded to a sub exponential growth pattern as already depicted in other rosette species [6]. Fitting an exponential model to this data revealed a similar behaviour for the Control and Below_PVPs treatments, with very close parameter values, whereas RER was significantly higher for plants grown between PVPs, specifically at early stages of development (Table 1 and Fig. 9b). Such an advantage, at early growing stages, for lettuces planted between two rows of PVPs was amplified until harvest due to the sub exponential growth model, resulting in the large differences observed in final leaf area.

**Table 1:** Parameters of the exponential growth model fitted for the 3 light treatments

	Control^a^	Below_PVPs^a^	Between_PVPs^b^
α	− 4.55	− 4.60	− 4.37
β	− 2.127 × 10^−3^	− 2.094 × 10^−3^	− 2.415 × 10^−3^

Relative Expansion rate (RER) was related to Plant leaf area with the following equation: RER = exp(α + β * Plant leaf area). Fitted lines are plotted in Fig. 9b. Different letters indicate significant differences between treatments (likelihood ratio test, α = 0.01).

#### Radiation Interception and Radiation Use Efficiencies in the field experiment

**Fig. 10:**
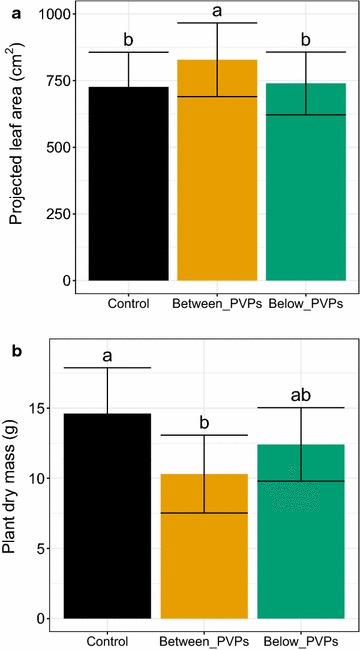
Projected leaf area and aerial dry mass determined at harvest for lettuce plants grown in field conditions with different light treatments (same notation as in Figs. 9, 10). **a** Mean projected leaf area at harvest (37 DAP). **b** Plant dry mass at harvest (37 DAP). Different letters indicate significant differences between treatments (ANOVA, Tukey tests, α = 0.05). Error bars indicate standard deviation for a minimum of 30 plants

The last images were captured at final harvest. As shown before throughout plant growth (Fig. 8b), final leaf area was significantly higher for plants grown between PVP strips compared to the other light treatments (Fig. 10a). Surprisingly, the plants grown between PVPs displayed the lowest dry mass (Fig. 10b).

**Fig. 11:**
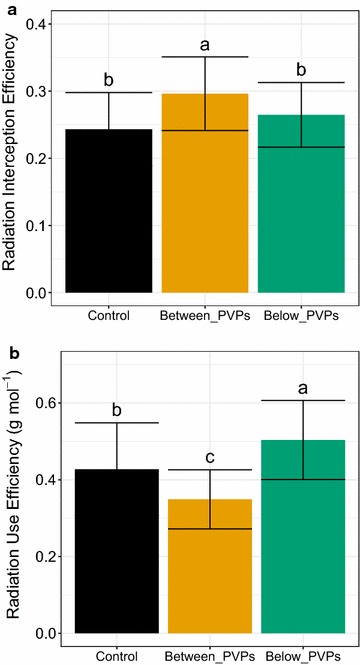
Radiation Interception Efficiency and Radiation Use Efficiency of lettuce plants grown in field conditions with different light treatments. **a** Radiation Interception Efficiency at harvest (37 DAP). **b** Radiation Use Efficiency at harvest (37 DAP). Different letters indicate significant differences between treatments (Kruskal–Wallis tests, α = 0.05). Error bars indicate standard deviation for a minimum of 30 plants

Radiation interception efficiency (RIE) and radiation use efficiency (RUE) were computed as the means for the whole growth cycle. As typically observed for shaded plants, RIE of plants grown in the two PVP treatments tended to be higher than Control plants. However, this gain in RIE was more marked and significant for Between_PVPs than Below_PVPs treatment (Fig. 11a) resulting from higher values of Plant Leaf Area. The 3 light treatments also induced significant differences in RUE (Fig. 11b), with the lowest values of RUE for plants of Between_PVPs treatment (Fig. 11b). On the opposite, Below_PVPs plants showed the highest values of RUE. This indicated that plants from this latter treatment, although with similar leaf area to control plants, tended to better convert intercepted radiation into biomass.

## Discussion

### A low-cost, flexible method for high throughput phenotyping of plant leaf area

The method presented herein to monitor projected, leaf area complies with a series of objectives aimed at facilitating high throughput applications in unstable environments. It is affordable, flexible, accurate and stable across variable light and background conditions.

Accuracy and stability of the method rest on 3 key developments. First, rather than focusing on image analysis, spectral specificities of the plant and background have been considered to adapt a low cost, standard camera where the infrared filter was removed and another one was added, transmitting only blue and near-infrared wavelengths. The resulting, new source image was much more appropriate for plant segmentation from the background since only plants have the ability to absorb blue and reflect infrared wavelengths. Inclusion of NIR wavelengths to detect leaf area was inspired by the widely used index NDVI [30] and is available or can be adapted with a large range of cameras. Contrary to analyses based on green detection in VIS images, emission of NIR wavelengths by leaves makes the method much less sensitive to different hues of green which may be due to nutrient status or genetic characteristics. Chlorotic spots in the vegetative area or even changes in leaf pigments with senescence or stress conditions could be detected in the NIR with our method. By contrast, green emission is typically dominated by other wavelengths in senescent leaves which preferentially degrade chlorophyll over carotenoids [51]. Analysis of VIS images in such cases typically requires adjustment by the user [52]. It is also possible to detect a mixture of leaves or plants within a unique image even with spectral variability in green wavelengths emission.

A second originality holds in the addition of the blue filter to better discriminate between plant and background which remains a major challenge [53, 54]. Most backgrounds reflect more blue light than do the plants. This specificity was implemented in our software analysis where, for each pixel, BLUE channel intensity was partly subtracted from RED channel one (mainly composed of NIR and IR wavelengths). As a result, a contrast could be found between plants and background in a large range of conditions using a unique script with stationary parameters for all image analyses. The value of “k” used in this manuscript to weigh BLUE values relative to RED ones was empirically optimized as a first approach, showing good correlation between manually and automatically estimation of plant leaf area. This correlation was hardly sensitive to variations of k between 0.4 and 0.6 when using our set of images. Mathematically optimizing the value of “k” by using a much larger set of images from different users could probably improve the method.

Last, the hardware that we proposed is based on light, small sized and affordable (low cost and widely spread) materials. We used a Raspberry Pi computer for its small size, low power demand and very low cost (5$–25$). As a computer, it is fully programmable so that image capture can be controlled in multiple, flexible ways, for example with different time lapses. The camera (Pi NoIR camera) is also very cheap (25$) with a relatively high resolution sensor (5–8 Mega Pixels), bringing to 50$–75$ the total cost of the imaging including cabling and storage. The whole device can be easily replicated to increase phenotyping throughput when plants are not potted or cannot be moved to the sensor. It can be adapted to multiple types of crops or canopies provided that specific holders are developed, going from simple terrestrial tripods to drones. For field application on medium sized plants, we have adapted a phenotyping cart (approximately 200$) where several “infrared” cameras were paired. About 2000 pictures of lettuce plants could be captured in about 2 h. Image analysis was then batch processed and took only a few minutes to obtain segmented images and estimation of plant area in pixels and cm^2^. With 6 cameras running in parallel, the phenotyping cart drastically reduced the time needed for image capture and permitted to estimate leaf area of plants difficult to access, with minimal disturbance of soil surface. The distance between the ground and the cell of the camera remained constant during the different experiments allowing for stable calibration of the cameras although a graduated gauge could also be incorporated in the field of view. Moreover, by using accessible and adaptable codes in the PYM method, we provided future users with enough flexibility to customize image capture and storage depending on their specific experimental design. For example, the PYM method already proved itself in PHENOPSIS platform.

The PYM method was developed to segment the plant from its background in challenging situations (field experiments). The only one trait we were interested in for lettuce was the projected area. But the method can now be plugged to another algorithms able to measure additional growth features, bases on high quality segmented pictures.

### Benefits of the method for field experiments

Devices able to monitor dynamics of plant leaf growth at high throughput are critically needed for agronomical or breeding purpose. This derives from the widely used approach of Monteith [55] which places leaf surface as limiting for light capture at the centre of the analysis when exploring differences between species or growing conditions.

We used this approach to explore the possible benefits of agrivoltaic systems where photovoltaic panels are combined with crops on the same land surface. Although the panels reduce the available light at the plant level, plant acclimation to shade can partly compensate for this limitation [41]. This point was confirmed for the Below_PVPs treatment in our experiment where plant biomass was much less reduced (by about 15% compared to control) than mean daily radiation available at plant level under the PVPs (reduced by 34%). This indicates that plants acclimated to conditions below PVPs to make a better use of available radiation than control plants. Acclimation of shaded plants was even more marked for leaf area which was enhanced for the Between PVP treatment (by 14%) while mean daily radiation available at plant level was reduced by 30%. Shaded plants are able to intercept more light with more elongated and thinner leaves, thereby counterbalancing the reduction in available radiation [56]. A previous study also reported higher RIE for plants shaded by PVPs compared to full sun conditions while RUE remained similar for all conditions [41]. However, this absence of significant difference in RUE was mainly due to a large heterogeneity between plants that was also observed in our experiment. We solve this difficulty by developing a phenotyping cart allowing for high throughput imaging which increased the power of statistical tests. Thus, significant differences between light treatments were not only detected for projected leaf area and RIE, but also in RUE contrary to the previous study [41]. Specifically, RUE was higher for lettuces grown below PVPs compared to full sun conditions, while RUE was lower for plants grown between panels. As a result of these differences in RIE and RUE, Below_PVPs plants were the most efficient as regards radiation use for biomass production, displaying intermediate plant dry mass at harvest compared to control and Between_PVPs plants. This conclusion could guide future developments of agrivoltaic systems.

The difference which was revealed between lettuces grown below the vertical projection of PVPs and under free spaces separating strips of PVPs was not expected since lettuces received quite similar levels of cumulated radiation at the whole day scale in both positions. Rather, we expected plants from Between_PVPs treatment to have accumulated more biomass due to their higher intercepting leaf area compared to plants grown at the vertical below PVPs. Furthermore, a slightly higher incident radiation at plant level for Between_PVPs treatment should have resulted in even higher biomass accumulation compared to Below_PVPs treatment. The lower dry mass at harvest for plants grown between PVPs more likely resulted from their lower RUE. In both treatments under PVPs, due to the South to North orientation of the plantation rows and the PVP strips, each lettuce was submitted to alternation of shade and full sun conditions with a different timing depending on the distance separating the plant from the vertical of the PVP strip axis. In our experiment, up to 1 h offset separated shading of the different rows depending on their position respective to PVPs. As a result, some rows may have been shaded when evaporative demand was maximal, notably those located below PVPs while Between_PVPs plants were exposed to full sun radiation and high evaporative demand which may have transiently limited growth and reduced RUE in this treatment.

Higher RIE and RUE in shaded plants was critical for maintaining production below PVPs. Increase of RIE in shaded conditions has been reported as the result of a higher intercepting leaf area [41]. In our experiments, striking differences in final plant leaf area were obtained between light treatments although with only slight differences in relative expansion rate at the beginning of plant development. This is typical of processes that follow exponential or even sub-exponential increase [48]. It has to be noticed that estimation of leaf area was only based on horizontal photographs, and it is not known whether vertical development of plants was also modified under PVPs to help maximising light interception [55]. Leaves typically get erected upon shading in most species but this probably has lower impact on light interception in oak leaf lettuce which exhibits very tortuous leaves, evenly oriented in all directions. An alternative explanation for the observed rise of RIE in plants shaded between PVPs is a possible increase of their specific leaf area, another typical response to shade in most species which reduce their leaf thickness to maximise leaf area per unit biomass. This may occur at the expense of RUE when the photosynthetic components per unit leaf area become limiting. This is a possible cause for the reduction in RUE observed in plants grown between PVPs which also exhibited the highest RIE in our experiment. Finally, it remains intriguing how plants grown below PVPs displayed higher RUE although higher photosynthesis efficiency has already been reported in shaded plants to better value intercepted radiation into biomass increase [56].

### Perspectives

Being based on spectral properties of leaf pigments and leaf cell structure, the presented method is relevant to all the plant species that we tested. Like for all other methods, weed control is very important to ensure that infrared reflection is only associated to the plant of interest. However, a combination of the PYM method with a more classical analysis of VIS images could possibly help discriminating between undesirable and targeted plants. Whenever possible, any undesirable materials (pots, sensors etc.…) in the field of view should preferably be selected as non-reflecting for NIR. Imaging environments are sometimes depleted in IR source light which can also be circumvented by adding an artificial source of IR light like LEDs illuminator (at a fixed wavelength, for example at 750 nm). Although not presented here, the procedure should be easily extrapolated to side-view images.

Finally, Raspberry Pi computers offer enough flexibility to fit scripts to multiple applications. For example, the system can be used for remote sensing of plant growth even during the night, using infrared LEDs programmed to light up during image capture. To save memory during image capture, the script developed for image analysis could be uploaded in the Raspberry Pi so that leaf area only could be stored. Segmented images could also be easily controlled by adding a portable display. The PYM method could thereby be appropriate for very large applications or drone assisted ones were the weight of the whole device should be minimized. However, high storage capacity devices are now available at low prices and low weight and are more flexible for further analysis.

Geopositioning of recorded pictures clearly extends the possibilities of plant phenotypic analyses. This is exemplified in the field experiment reported herein where moving the phenotyping cart along a predefined pathway and following a sequential naming of the pictures, made possible to automatically locate each recorded image with respect to PVP shading. Spatial effects could thus be tested and a difference was revealed between lettuces grown below the vertical projection of PVPs and below free spaces separating strips of PVPs. Similar procedure could apply to the analysis of any spatial structure that could have influenced crop growth like soil heterogeneity, distance from neighbouring trees in agroforestry or distance to drippers or sprinklers in irrigated systems.

## Conclusions

We elaborated a new imaging device associated with a robust image analysis routine to estimate plant leaf area in a large diversity of environments. The method took advantage of the spectral properties of leaves emitting in infrared wavelengths. The hardware was developed around the widely used Raspberry Pi computer and camera, resulting in a very low-cost device. Pairing several devices together, high throughput could be reached to reveal subtle differences in leaf growth when conditions induce scattering in plant growth. An application is presented in field conditions where the method revealed acclimation of lettuces plants to shading by photovoltaic panels via modifications in RIE and RUE. Low-cost, light maintenance and flexibility of the method can meet a growing demand for plant phenotyping with multiple purpose.

## Additional files

**Additional file 1.** Photograph of the phenotyping cart operating on field grown lettuces. Plantation boards were composed of 6 rows. Each row was associated with a single camera, resulting in 6 cameras mounted on Raspberry Pi and attached to the cart at 1 m height above soil level. Two operators, one on each side of the crop plot, moved the cart row after row, one operator triggered the image capture. A, B and C: side views of the phenotyping cart. D: Wiring between contactor and one Raspberry Pi. E: Contactor triggering the image capture on each camera. F: Wiring at the 1st Raspberry Pi level, supplying power for image capture triggering. G: Detail of wiring at the pin levels for the 1st Raspberry Pi. H: Detail of wiring at the pin levels for all others Raspberry Pi. I: Blue filter glued to a camera.

**Additional file 2.** Detailed wiring between the contactor and Raspberry Pi computers. By default, GPIO18 is connected to Ground, when the contactor is pushed, electric power is transferred to GPIO18, launching a script in the Raspberry Pi, triggering the image capture on all connected devices. A 470 k Ω resistor was attached to the GPIO18 entry at the contactor level to reduce. The cameras are not shown in this view.

## Abbreviations

BF: blue filtered
IR: infrared
LED: light-emitting diode
NDVI: normalised difference vegetation index
NIR: near infrared
PVPs: photovoltaic panels
RER: relative expansion rate
RIE: radiation interception efficiency
RUE: radiation use efficiency
SLA: specific leaf area
TT: thermal time
VIS: visible
