# Supplementary figures and images for: PYM: a new, affordable, image-based method using a Raspberry Pi to phenotype plant leaf area in a wide diversity of environments
Source: Plant Methods. 2017 Nov 8;13:98. doi: 10.1186/s13007-017-0248-5 (PMC5678554; doi:10.1186/s13007-017-0248-5)

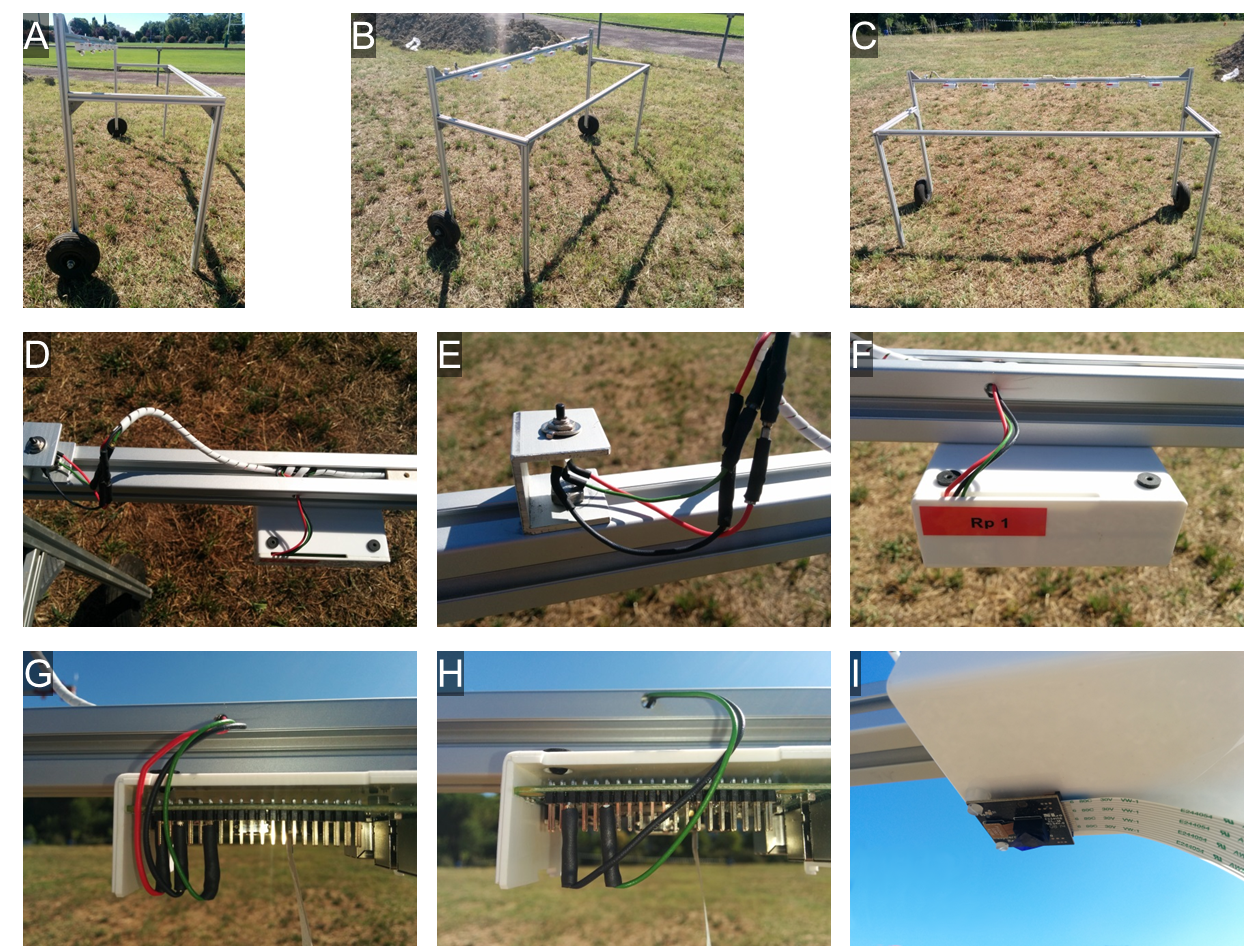

Supplement: Supplementary file 1 — Additional file 1. Photograph of the phenotyping cart operating on field grown lettuces. Plantation boards were composed of 6 rows. Each row was associated with a single camera, resulting in 6 cameras mounted on Raspberry Pi and attached to the cart at 1 m height above soil level. Two operators, one on each side of the crop plot, moved the cart row after row, one operator triggered the image capture. A, B and C: side views of the phenotyping cart. D: Wiring between contactor and one Raspberry Pi. E: Contactor triggering the image capture on each camera. F: Wiring at the 1st Raspberry Pi level, supplying power for image capture triggering. G: Detail of wiring at the pin levels for the 1st Raspberry Pi. H: Detail of wiring at the pin levels for all others Raspberry Pi. I: Blue filter glued to a camera. [file 13007_2017_248_MOESM1_ESM.png]
