# Supplementary material for: PYM: a new, affordable, image-based method using a Raspberry Pi to phenotype plant leaf area in a wide diversity of environments
Source: Plant Methods. 2017 Nov 8;13:98. doi: 10.1186/s13007-017-0248-5 (PMC5678554; doi:10.1186/s13007-017-0248-5)
Supplement: Supplementary file 2 — Additional file 2. Detailed wiring between the contactor and Raspberry Pi computers. By default, GPIO18 is connected to Ground, when the contactor is pushed, electric power is transferred to GPIO18, launching a script in the Raspberry Pi, triggering the image capture on all connected devices. A 470 k Ω resistor was attached to the GPIO18 entry at the contactor level to reduce. The cameras are not shown in this view. [file 13007_2017_248_MOESM2_ESM.pdf]

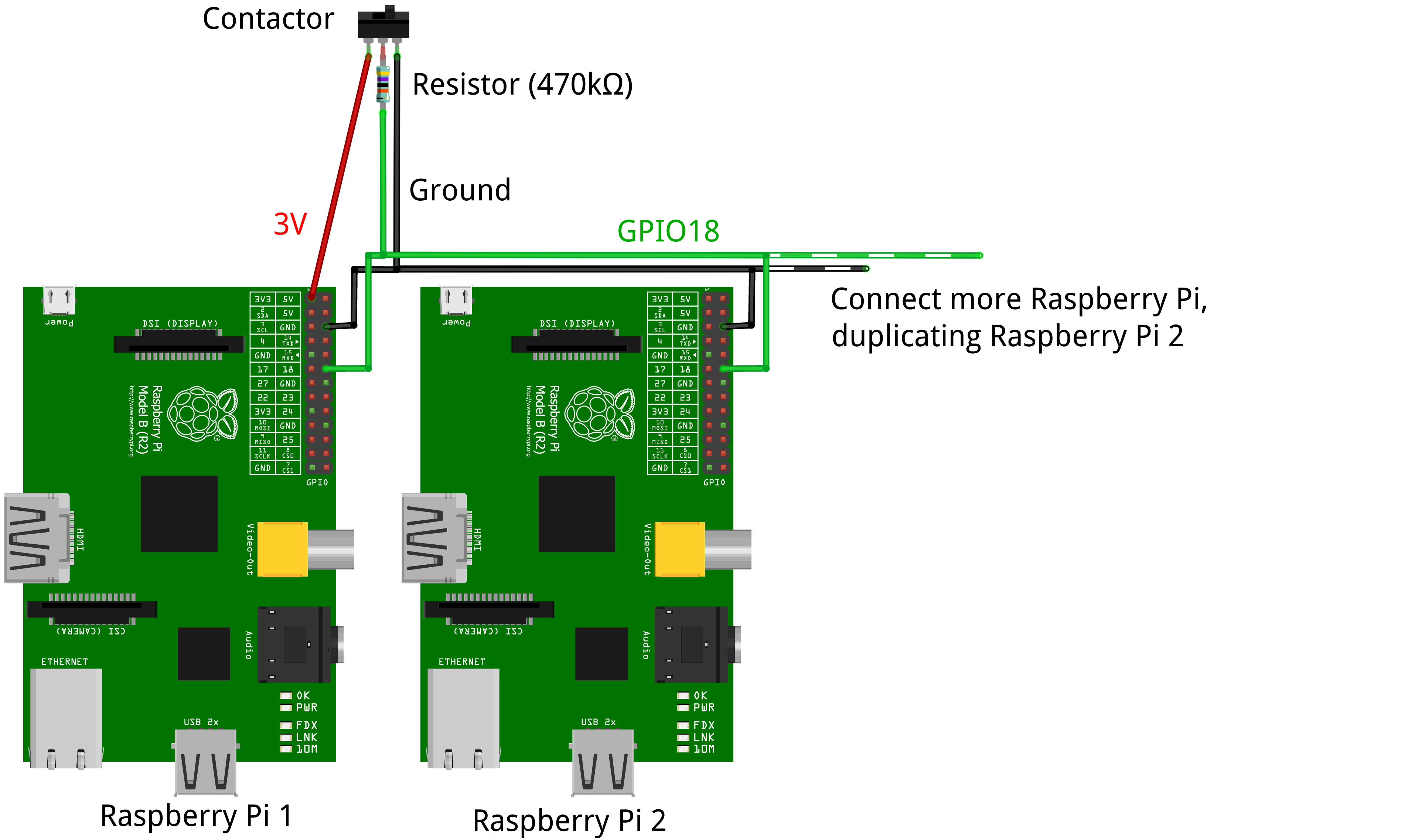
Contactor
Resistor (470kΩ)
Ground
3V
GPIO18
Connect more Raspberry Pi, duplicating Raspberry Pi 2
Raspberry Pi 1
Raspberry Pi 2
fritzing
